# The Criminal’s Plea of Irresponsibility1Read before the Buffalo Medical Club.

**Published:** 1895-11

**Authors:** James W. Putnam

**Affiliations:** Clinical professor of nervous diseases, University of Buffalo.


### THE CRIMINAL'S PLEA OF IRRESPONSIBILITY.[1]

By JAMES W. PUTNAM, M. D.,

Clinical professor of nervous diseases, University of Buffalo.

SINCE the acquittal of McNaughten, in England, on the ground of insanity, there has been an increasing humanitarianism on this subject. Formerly, only that form of mental disease which brought its victim to the level of an unreasoning wild beast, or of a driveling idiot, was recognised as an excuse for crime. Today, any form of mental disease can be brought forward as a plea for the acquittal of criminals. This is a fact as well understood by the criminal classes as by the legal or medical professions.

The criminal not only knows that insanity is a sufficient plea for acquittal, but, also, that public opinion is against holding an insane person responsible for crime. The criminal can judge from the details of medical testimony, published in the papers during the progress of all important trials, just what may be considered as bearing weight in coming to a diagnosis of insanity. Among other things, he learns that it is important to him to have all the family record quite familiar to him; that intermarriage of cousins as parents is not a bad starter; that an insane or epileptic uncle, or aunt, or grandparent is a good thing to remember; that epilepsy in the family is of value in proving a neuropathic tendency.

Alcoholism in father or mother is now to be brought into court as bearing upon the criminal's tendency or liability to insanity or to his neuropathic taint. Then, as to the personal history of the criminal, convulsions in childhood, cruelty to animals or to children, falls upon the head, tendency to headache—all these things are very important and must be remembered, and if they never occurred it is well to manufacture them, so as to have the detailed history ready for use on an emergency. The record of his boyhood, at school in sports if quarrelsome, if peevish, if given to going alone and not joining with others—all these things have, time and again, in different noted cases, been brought forward as connecting links in the chain of evidence for insanity. These things we know and the lawyers know, and before the trial the prisoner and the prisoner's family and friends know.

They know, also, that to hear voices at night, to be suspicious of one's friends, to have ideas that people are against him, to for-

1. Read before the Buffalo Medical Club.

get the details of a crime, all have weight with doctor and jury. With all this knowledge the criminal of the higher class, who is caught red-handed and who has no chance of escaping the penalty of the law by proving an alibi, or extenuating circumstances, pleads not guilty, but insane.

As public sentiment stands, criminals are acquitted today, by reason of a certain deviation from an ideal, moral or intellectual type, who would not have been acquitted ten years ago. Such cases are the paranoiacs, the victims of certain forms of melancholia and cases of psychopathia sexualis. Yet none of these are so mentally affected that they come under the ruling of English judges, which is, that the criminal cannot be held responsible if, at the time of the commission of the act, he was incapable of distinguishing right from wrong and did not know that the act was an offense against the laws of God and nature.

Lord Deas, of Aberdeen, charged as follows: "If the jury believed that the prisoner, when he committed the act, had sufficient mental capacity to know and he did know that the act was contrary to the law and punishable by the law it would be their duty to convict him."

Judge Smith, in Rochester, New York, charged: "A man must have sufficient knowledge, reason, capacity and mental power to understand not merely that his act is in violation of law, but that it is intrinsically wrong."

Recorder Hackett said: "By the state of insanity I am to be understood as meaning the state under which a man is not accountable for an alleged criminal act, because he does not know that the act he is committing is unlawful and morally wrong and has not reason sufficient to apply such knowledge and to be controlled by it."

This ruling is, to me, correct as far as it goes, but I think it does not go far enough. It does not include those undoubted criminals who know an act is wrong, but have no power of control. As, for example, the insane woman who murders the child she loves after having resisted the desire to kill it on previous occasions and finally yields to it.

Ordronaux includes this idea in his summing up of the questions to be determined in relation to criminal responsibility:

> Whether, knowing the nature and consequences of the act, he had the power to choose between doing and not doing it, and whether, supposing he had lost the power of choosing between right and wrong in reference to the particular act, he had lost that power through disease and not through intoxication, violent anger or any form of self-produced mental convulsion.

This last phrase—mental convulsion—I do not fully understand, as it is not sufficiently definite. These tests, it will be seen, would not excuse the act of Prendergast, who knew that murder was wrong, and who murdered to avenge a fancied wrong, although the evidence tends to show that he was insane prior to his crime. It does not include, nor do I claim it should include, the great majority of sexual perverts. Because one man has an unnatural lust for another, and because, later, his beloved leaves him, proposing to marry, if the pervert murders either him or his ladylove to soothe his wounded feelings, I see in that no excuse for crime, no evidence that he did not know right from wrong, no evidence that he could not have controlled himself, but that he *would not.* And, right here, I ask, is it not possible that the fact that the pervert knows that his failing is recognised by some as insanity and as an excuse for crime, for they all know this since the Alice Mitchell and Freda Ward case, that he may feel that it is not necessary for him to control himself? It has seemed to me that there is a certain danger in this feeling of security that many men must have, that, no matter what they do, they will be acquitted. Take, for instance, the criminal who has committed crime, who has had insane ancestors, and who himself has been adjudged insane at a former trial, and has afterward been discharged recovered from an asylum. Let him out in the world, and I believe he feels secure that future crimes will go unpunished, and I share in his belief. He, in all probability, would go unpunished, whether at the time of the commission of a crime he was sane or insane, because his former attack would be of great weight with the jury, and because he would know the value of his former delusions and hallucinations.

Take the case of the epileptic, the educated criminal, who is up in the history of crime and its defense, knows that the post-epileptic state is an irresponsible one, knows that the psychical equivalent for epilepsy is an excuse for crime, is a state of irresponsibility. I do not see why the epileptic is not quite free, in his mind, as to the consequences of any crime he may choose to commit, but I believe few juries would convict a well-defended criminal who was an epileptic, even if that epileptic knew right from wrong, and hosts of witnesses testified to his mental capacity. I have chosen to write this paper to point out some of the dangers of the present views of insanity. In common with others, I firmly believe in the irresponsibility of some lunatics. But I also believe that many lunatics commit crimes deliberately, with malice, for which they are responsible, and in which they are protected by science. There are some forms of insanity, such as mania, dementia, profound melancholia and chronic lunacy, which all agree are excuses for crime. But transitory mania, in which the man was sane up to the commission of crime, insane during the crime, and sane afterward, must be looked on with suspicion. Alleged delusions must be well proved not to be lies before they are accepted as proof of insanity. Because a criminal, arrested for theft, tells us he owned the store from which he took the goods, we must not necessarily infer he has a delusion. It is possible he lied. If a man murders another and, in jail, tells us calmly he did it because God commanded him to do so, we must remember that a sane man would gladly say that if it would clear him; or if he says the victim was one of a band of conspirators, it must be still further sifted. There is, in many cases, a tendency to believe that all the stock statements of lunatics are proofs of lunacy. Of course, this is not denying that they are not to be investigated and given their full value.

The question of drunkenness as a defense for crime has been summarised by Clark Bell from English and American laws:

While drunkenness is not, *per se*, a defense upon a charge for crime, yet mental unsoundness, superinduced by excessive intoxication and continuing after it has subsided may excuse; or where the mind is destroyed by a long-continued habit of drunkenness, or where the long-continued drunkenness has caused an habitual madness which existed when the offense was committed, the victim would not be responsible. For if the reason be perverted or destroyed by a fixed disease, although brought on by his own vices, the law does not hold him accountable. The rule of law is well settled that evidence of intoxication is admissible to explain the conduct and intent of the accused in cases of murder. This rule does not apply to other crimes where the intent is not a necessary element to constitute a degree or phase of the crime.

In cases where the law recognises different degrees of a given crime and provides that willful and deliberate intention must be actually proved to convict of the first degree, it is a proper subject of inquiry whether the accused was in a condition of mind to be capable of premeditation. The reason of this is that the fact of intoxication is an aid to determining whether the act was the result of deliberate and malicious intent and because, as the New York penal code says, section 22: No act committed by a person while in a state of intoxication shall be deemed less criminal by reason of his having been in such a condition. But whenever the actual existence of any particular purpose, motive or intent is a necessary element to constitute a particular degree of crime the jury may take into consideration the fact that the accused was intoxicated at the time.

There has developed, in late years, a class of citizens who are inclined to quarrel with this law. Notable among these is Dr. T. D. Crothers, who recently testified in Buffalo on a murder trial. He said, in his opinion, that the effects of one drink of whisky upon the brain would never be removed, and testified, in substance, that any man who had ever taken alcohol was not responsible. This did not appeal to Buffalo jurymen, and the criminal was convicted. Dr. Crothers, with a numerous following, is striving to have crimes committed by drunkards placed in the same category as crimes committed by the insane. From my reading of this subject, this seems a most dangerous position, so far as society is concerned. Were such a theory to hold, we could only punish criminals who were total abstainers, or, at least, very moderate drinkers, a class not common these days, and would be very rare indeed if all that were necessary to plead not guilty with success was to have a record of a few previous intoxications.

I have thus outlined many of the points which arise in the criminal's view of the plea of not guilty because irresponsible, not so much to impart new ideas as to provoke discussion.

388 Franklin Street.

